# Mumps

**Published:** 1908-10-24

**Authors:** 


### Diseases of Children.

### MUMPS.

Mumps is generally regarded as a trivial complaint. In young children it is quite exceptional for serious complications to occur or for any destructive effects to be produced by this infection. For this reason the disease is often regarded with amusement, or even contempt, until the neglect of simple precautionary measures has ended in dangerous complications or permanent damage to the body. Although the disease has been reported in the new-born, it is rare in early childhood, and infants are remarkably immune. It is most common from the fourth to the fifteenth years of age, during the period of school life, when it is likely to assume epidemic prevalence.

In type mumps varies considerably in different epidemics. At present there is a mild variety very prevalent in a north-country health resort, and, as usual in these mild epidemics, there are few complications. The incubation period is from 14 to 21 days, but cases have occurred even as late as the thirty-third day after exposure. Quarantine should be maintained for three to four weeks after exposure, but it need not be enforced during the first half of the time. In some cases, the attack is ushered in with vomiting, high fever, and even a rigor or convulsions. Gastro-intestinal symptoms may be marked. More often there is merely a little malaise, with stiffness of the jaws, difficulty in opening the mouth, pain on eating, dulness of hearing, pricking in the ear, and stiffness of the neck. The pain is most marked in the temporo-maxillary articulation, and, like the swelling, is often unilateral. Sometimes there may be no discomfort throughout the attack, although there is marked swelling.

It is to the complications, however, of this disease that particular attention should be directed, especially to those affecting the testicles, ovaries, mammæ, and pancreas. Of these, orchitis is in some respects the most important. It is quite exceptional under twelve years of age. Its frequency varies in different epidemics. It begins at the end of the first or beginning of the second week, and lasts for about a week. Dukes states that it almost invariably begins on the seventh or eighth day. It may begin on the third or not until the tenth day. Sometimes it is primary, the salivary glands being unaffected, and the causation is then liable to be overlooked unless mumps is epidemic. In these rare primary cases, the onset may be acute and the attack lasts for two to three weeks. They are said to occur only in adults. The orchitis may be accompanied by high fever and delirium. It is of grave importance, for it may be followed by atrophy of the testicle: fortunately it is generally unilateral. In view of the fact that it is rare after the eighth day, it is evident that, as a precaution, boys of twelve years or more of age should be kept in bed for at least eight days.

In girls, occasionally in very young ones, ovaritis and mammary swelling have been noted. Occasionally the labia majora are swollen. Troitsky (*La Pediatria*, p. 241, 1906) found 13 out of 33 girls affected. In eight of them the catamenia had appeared. The ovaritis, or öophoritis, was unilateral or bilateral, according as the mumps was unilateral or bilateral. Atrophy, and parenchymatous or even interstitial changes in the gland, may follow and lead to sterility. Cold applications and rest in bed should be adopted.

Metastatic pancreatitis is important and may be dangerous. So far, it has been reported chiefly in adults, *e.g.*, 10 out of 652 soldiers (Simonin, 1903); a fatal case in a soldier, aged 19 (Lemoine and Lapasset, 1905); in 20 out of 26 cases (Cuche). But it is by no means improbable that such cases occur equally often in children, and that they would be diagnosed if the complication were suspected. A few have been recorded at 10 years of age. Usually it is mild, and comes on about the third to the sixth day, and lasts for two to seven days. Pain is felt in the epigastrium and left hypochondrium, with tenderness on pressure. Deep palpation is impossible. Nausea and vomiting are rarely absent. Diarrhœa occurs in half the cases; but in Edgcombe's cases constipation was present. Fever is variable and may be absent. Blood and fat may be found in the stools. There is no glycosuria. Acetone and diacetic acid have been found in the urine, and Cammidge's pancreatic reaction may be present in the urine, as well as a marked deposit of calcium oxalate crystals. The swelling of the pancreas may be felt if there is no undue tenderness and no abdominal distension, or it may not be palpable until the tenderness has subsided. There is no evidence of subsequent atrophy. Tenderness over the pancreas may be the only indication. The fatal case followed a mild attack of mumps. On the fifteenth day there developed frequent vomiting, epigastric tenderness, prostration, and bradycardia. These symptoms were followed by progressive jaundice, hæmatemesis, anuria, constipation, unconsciousness, and death on the seventeenth day.

Meningitis has occurred in adult males. Bradycardia, headache and fever are the main symptoms. An excess of lymphocytes is found in the blood and in the cloudy cerebrospinal fluid. Lymphocytosis is a relative and absolutely constant symptom of mumps from the onset until the swelling has gone, more marked in bilateral than in unilateral cases and in children at puberty (Wile: *Archives of Pediatrics*, 1906). It may afford a valuable diagnostic point between mumps and adenitis. Meningitis, or meningo-encephalitis, is more common than is supposed. It appears to be limited to the region of the medulla and pons, and probably accounts for some of the occasional complications and sequelæ, such as delirium, headache, optic neuritis, facial palsy, deafness, herpes of the fifth nerve, Kernig's sign, and bradycardia.

Deafness may be unilateral, permanent, and unassociated with otitis media; it may be due to otitis media; or it may be caused by labyrinthitis, coming on abruptly, and even preceding the swelling of the parotid. Optic neuritis has been recorded in adults and is usually recovered from, but may end in atrophy. Iritis and paralysis of accommodation occur. Nyctalopia has been reported in a girl of 16 (Campani, 1903). Facial palsy may result from pressure by the swollen gland. Endocarditis is very rare; but Tatschner, in 1904, noted four cases in one family. Myocardial degeneration with cardiac dilatation is more likely to occur. Still rarer are acute nephritis, swelling of the lachrymal gland, thyroid and thymus, temporary dementia and loss of memory. Nephritis begins during convalescence, almost always becomes hæmorrhagic in type and is usually benign. It may become chronic and end fatally.

This formidable list of complications shows that mumps is by no means a trivial and unimportant disease. Fortunately the attack is usually uncomplicated, especially in children. It is, however, of the utmost importance that great care should be exercised in the treatment of the disease in boys and girls from the age of ten onwards, and that a lookout should be kept for orchitis, oophoritis, and pancreatitis. Rest in bed is the most important point in the treatment.

